# Decision Support System in the Field of Defects Assessment in the Metal Matrix Composites Castings

**DOI:** 10.3390/ma13163552

**Published:** 2020-08-12

**Authors:** Robert Sika, Michał Rogalewicz, Paweł Popielarski, Dorota Czarnecka-Komorowska, Damian Przestacki, Katarzyna Gawdzińska, Paweł Szymański

**Affiliations:** 1 Division of Foundry, Institute of Materials Technology, Faculty of Mechanical Engineering, Poznan University of Technology, Piotrowo 3 Str., 61-138 Poznan, Poland; Robert.Sika@put.poznan.pl (R.S.); Pawel.Popielarski@put.poznan.pl (P.P.); Pawel.Szymanski@put.poznan.pl (P.S.); 2 Division of Production Engineering, Institute of Materials Technology, Faculty of Mechanical Engineering, Poznan University of Technology, Piotrowo 3 Str., 61-138 Poznan, Poland; Michal.Rogalewicz@put.poznan.pl; 3 Polymer Processing Division, Institute of Materials Technology, Faculty of Mechanical Engineering, Poznan University of Technology, Piotrowo 3 Str., 61-138 Poznan, Poland; 4 Division of Machining, Institute of Mechanical Technology, Faculty of Mechanical Engineering, Poznan University of Technology, Piotrowo 3 Str., 61-138 Poznan, Poland; Damian.Przestacki@put.poznan.pl; 5 Department of Machines Construction and Materials, Maritime University of Szczecin, 2–4 Willowa St., 71-650 Szczecin, Poland

**Keywords:** metal matrix composites, casting defects, classification, defects atlas, open-access application

## Abstract

This paper presented a new approach to decision making support of defects assessment in metal matrix composites (MMC). It is a continuation of the authors’ papers in terms of a uniform method of casting defects assessment. The idea of this paper was to design an open-access application (follow-up system called Open Atlas of Casting Defects (OACD)) in the area of industry and science. This a new solution makes it possible to quickly identify defect types considering the new classification of casting defects. This classification complements a classical approach by adding a casting defect group called structure defects, which is especially important for metal matrix composites. In the paper, an application structure, and the possibility of its use in casting defects assessment were introduced.

## 1. Introduction

Metal composites are increasingly replacing traditional materials used in construction, aviation, and in the construction of machinery and equipment and in many other fields. This is due to the ability to obtain virtually any set of desirable functional properties of the material, such as a high damping factor, high resistance to abrasion, high Young’s modulus, low specific weight, and low coefficient of thermal expansion. According to Konopka at el., the definition of composite material presented in publications [1,2,3] is as follows: “Composite material is created by tightly binding at least two chemically and physically different materials together in such a way that, while maintaining a good and permanent connection of the components, a clear boundary is maintained between them and that the distribution of reinforcing components throughout the entire matrix volume is as uniform as possible”. This definition describes an ideal composite material with a perfect structure. Real composite materials generally have imperfect structures—composite materials contain various defects [1,2,4,5,6,7,8,9,10,11,12], especially when considering cast composites. Castings feature a specific structure that is related to the course of the manufacturing process [13,14,15,16,17,18,19,20,21,22,23,24]. The classification of these irregularities makes it possible to:

- Precisely identify them;
- Determine why they form;
- Determine which manufacturing stage causes their formation;
- Promptly take countermeasures.

There is no such classification in the case of metal composite castings. Essam M. [25] confirms this, presenting the surface defects of composites with aluminum matrix and Al_2_O_3_ reinforcement, and especially the assessment of the distribution homogeneity of the reinforcing phase [25] and macroscopic studies. No scheme or key describing irregularities in the structure of these materials was used. The authors of this paper meticulously, but without confirmation with any standard, intuitively identified and interpreted all defects, which caused some problems in the reception of the publication. In contrast, Mamalis et al. also documented defects that occurred during the processing of composite materials (e.g., the fiber-reinforced materials, metal matrix composites, ceramic matrix composites and bonded materials) [26]. Although the work is a valuable supplement to knowledge related to the alleged mechanism of reliability of composites during their useful life, there are no clear terms enabling the efficient assessment of the work of the material by a diagnostician or an operator. On the other hand, the classification of defects in castings of traditional materials (cast iron, cast steel, non-ferrous metal alloys) is insufficient and must be supplemented with specific defects of those materials. This problem, which was noticed during the description of the structure and quality of metal composite castings, was the reason for undertaking works on the development of such classification and, as a result, for this work to be created. This paper presented a new approach to decision making support of defects assessment in metal matrix composites (MMC). It is a continuation of the authors’ papers in terms of a uniform method of casting defects assessment. The idea of this paper was to design an open-access application (follow-up system called Open Atlas of Casting Defects, OACD) in the area of industry and science. Simulation codes currently used in the foundry industry are primarily used to predict casting quality, quality tied mainly to the location of defects such as shrinkage (voids of shrinkage origin). Prediction of zones exposed to other casting defects (i.e. erosion of mould, the presence of non-metallic inclusions, zones exposed to "hot tears", to penetration of the mould by the liquid alloy) takes place on the basis of models –empirical formulas (called soft) or indirectly on the user’s knowledge and analysis of the results of simulation, for example, speed field of metal stream in the mould cavity or the time-temperature image of cast and mould interaction. Other modules used in foundry simulation codes taking into account the diffusion of alloying elements (Fick’s equation) or thermomechanical processes enabling modeling of stress in a casting-mould system using constitutive equations. These activities are the basis for decisions concerning the selection of the optimal casting technology with the expectation of obtaining the final, acceptable version of the concept, taking into account the criterion of the best relationship of quality/price of the casting. This made it possible to eliminate, commonly used over the years, classical method of trial and error in the design process of casting technology, completed only through intuition of engineers, specimen castings and experimental tests. The technologists with simulation system and with the results of calculations using this tool, take decision on the basis of their assessment and based on the above, then suggest the next version after the change of technology/casting design or approve designed technology.

The scope of the paper includes the classification of defects, taking into account foundry iron alloys, i.e., alloy of iron with carbon up to 1.5% C in the cast state—cast steel and an alloy of iron with carbon from 2.11–4.3% C in the cast state—cast iron, and non-ferrous metal alloys, mainly aluminum with silicon—silumines and metal composites’ liquid matrix technologies (direct methods—in situ: production of composites by mechanical mixing and indirect methods—ex-situ: production of composites by mechanical mixing and production of composites by saturating the porous structure with liquid matrix metal).

## 2. Classification of Metal Matrix Composites

### 2.1. Division of Composite Materials

Composite materials can be divided depending on the production method, intended use, technological properties, and depending on the matrix as well. Metal matrix composites are currently produced with a variety of methods, which include but are not limited to [2,3,27,28,29,30,31,32,33,34,35,36,37,38,39,40,41,42,43,44,45,46,47]:

- Liquid matrix technologies (only these will be the subject of this paper because it concerns defects of castings);
- Deformation technologies, i.e., those that use plastic processing methods;
- Sinter technologies.

In the liquid matrix technology, there are two fundamentally varying methods of composite production, such is direct methods, and indirect methods [2,3,8,28,30,32,37,38,44,45,46,47].

### 2.2. Direct Methods in Which the So-Called “In Situ” Composites Are Made

In situ composites belong to the group of composite materials in which the reinforcing phase is formed in the matrix material in the composite production process. In this process, it is possible to obtain reinforcement with various structures and properties. The reinforcing phase can be in the form of dispersion particles or fibrous phases; it can be ductile or brittle.

**Figure 1:**
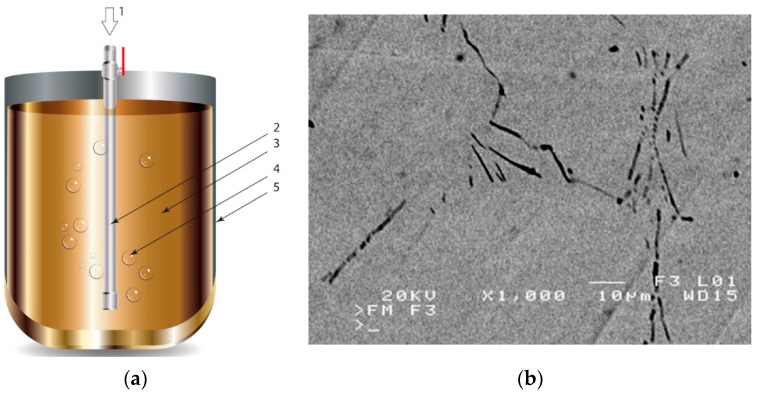
Production of a composite in a liquid—gas system: (**a**) scheme: 1—gas, 2—gas lance, 3—liquid metal, 4—gas bubbles, 5—crucible; (**b**) SEM image of the in-situ Al_3_Mg with N_2_ composite.

There are many methods for obtaining this type of composite [2,3,32]. Reinforcement may occur in processes involving the liquid phase (Figure 1) or the solid phase. The production of reinforcement from the liquid phase can occur in the process of crystallization, for example, in directional crystallization of eutectic systems, by strong gas supercooling of supersaturated solutions or a rapid reaction between components in the liquid phase. Processes using internal oxidation, substitution reactions or reactive component grinding are methods of producing in situ composites with the participation of the solid phase [2,32].

### 2.3. Indirect Methods, Called Ex-Situ Methods

**Figure 2:**
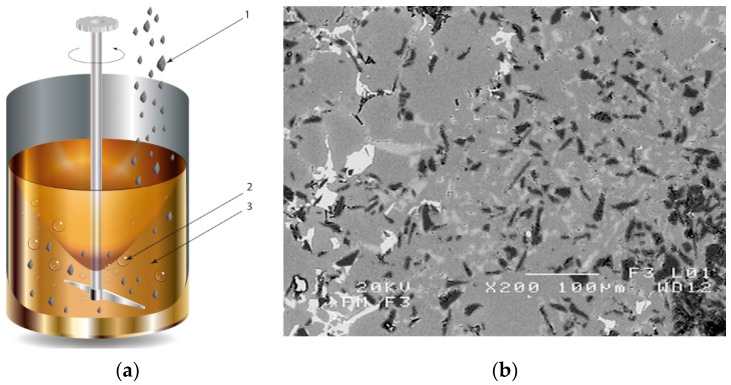
Production of composites by mechanical mixing: (**a**) scheme: 1—particles-reinforcing phase, 2—gas bubbles, 3—liquid metal; (**b**) SEM image of the ex-situ composite microstructure (suspension composite: matrix—Al alloy, reinforcement—SiC particles).

In the case of these composites, castings are obtained in two ways. The first method involves mixing the liquid matrix, which is a technical alloy (Figure 2) with fixed reinforcement (suspension composites) [2,3,41,45]. The composite suspension is most often obtained by introducing ceramic particles to the liquid alloy matrix: during the mechanical mixing, by dissolving the composite concentrate, blowing ceramic particles using gas or ultrasonic or electromechanical mixing.

Light alloys such as aluminum, magnesium, lithium, titanium as well as copper, iron and their alloys are most commonly used for the matrix. The reinforcing phase is constituted by graphite, glass, aluminum, silicon, zirconium, titanium, cerium oxides, as well as silicon and titanium carbides or boron nitride. The reinforcement is in the form of particles with granularity ranging from a few to several hundred micrometers or fibers with a diameter of 0.2–4 mm and length of 0.5–2.0 mm. Its concentration in the matrix reaches 30%.

**Figure 3:**
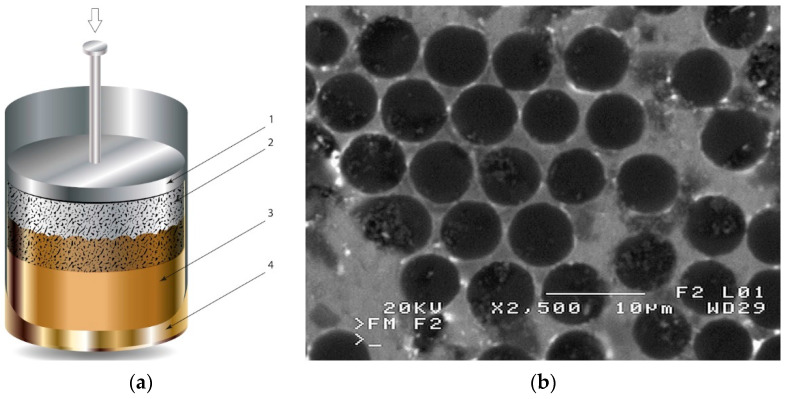
Production of composites by saturating the porous structure with liquid matrix metal: (**a**) scheme of the mould together with a moulder—preform during saturation, 1—stamp-piston, 2—ceramic moulder partly saturated with metal, 3—metal, 4—metal sleeve; (**b**) SEM image of the composite boundary with saturated reinforcement (Al alloy with C fibers).

The second method involves saturating the porous structure of the composite reinforcing phase with a liquid technical alloy (composites with saturated reinforcement), most often under pressure [1,2,3,8,38], Figure 3. Various metals and their alloys can be used as the matrix. Due to manufacturing difficulties, casting temperature and activity in relation to the environment may be a barrier to their use. For these reasons, aluminum and magnesium alloys, some copper alloys and, to a lesser extent, low fusible alloys, are mainly used. The reinforcement of saturated composites can be constituted by metal materials, e.g., carbon and alloy steels, and non-metallic materials, e.g., ceramics (aluminosilicates, carbon-graphite), boron, polymeric materials [2,3]. These materials may be in the form of structured long fibers, yarn, fabric, mat, unstructured short fibers, cotton wool, wool, cellular structures, sinters, etc.

## 3. Classification of Defects in Castings of Various Metal Materials

The factor determining the procedure in the casting production process is the request (order) of the recipient which specifies their needs regarding the casting [1,48]. This factor affects further proceedings. The quality of castings is affected, among others, by factors related to the requirements specified in the order, as well as strictly technological factors such as casting process design, casting materials and metal melting, mould filling with metal, solidification, crystallization, cooling and removal of the casting from the mould.

Failure to meet the above requirements may result in the formation of defects in castings. All metal castings have defects of various types and origins. Any deviation in the characteristics, structure and mechanical or physicochemical properties of a material from the applicable requirements can be called a casting defect or defects [13,16,48,49,50,51,52,53,54,55,56,57,58,59,60,61,62]. Defects can be identified based on their features, which in turn leads to the creation of the casting defect classification. This classification is useful for:

- Transfer of information in research work, during the educational process, or in the manufacturing process;
- Elimination of defective castings from further stages of the manufacturing process;
- Intervention activities aimed at removing the causes of defect formation from the manufacturing process.

Regarding the second case, a classification criterion of defective castings is a division of castings into three casting groups [13,16,61,62,63,64,65,66,67]:

- Satisfactory castings with acceptable defects;
- Castings with repairable defects;
- Castings with disqualifying defects.

For castings made from traditional materials, there are standards, atlases, or catalogues of defects [13,16,27,57,61,62,63,64], which:

- Enable unequivocal identification of defects;
- Provide methods to detect them;
- Provide causes of their formation;
- Suggest technological means to prevent their formation.

**Figure 4:**
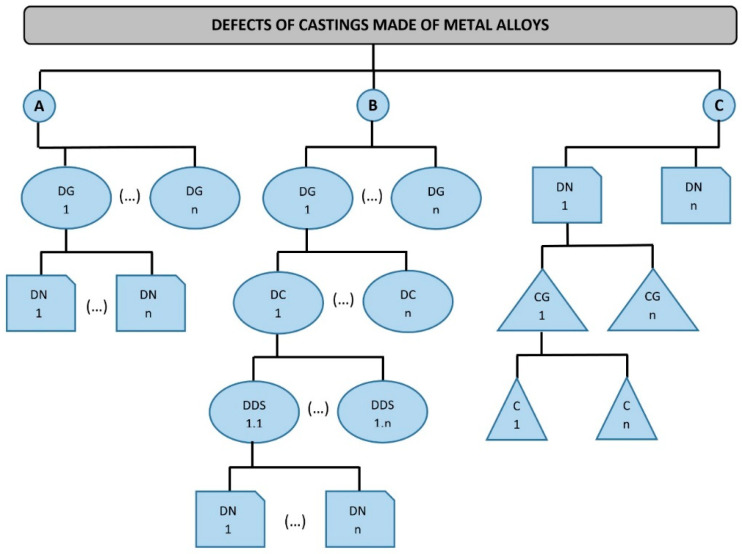
Classification schemes of defects in castings from traditional materials (own elaboration on the basis of references [27,57,60,61,63,64,66]): A—according to Polish standards, B—according to French standards, C—according to the English and German systems, DG—defect group, DN—defect name, DC—defect characteristics, DDS—more detailed defect specification, CG—cause group, C—cause.

They also include numerous publications that identify, describe or detect defects in castings. Zhao et al. presented a defect description system based on the Radiographic Images and Sparse Representation-based Classification (SRC) [65]. However, only four popular types of casting defects, including cracks, blow holes, shrinkage porosities and shrinkage cavities, were considered in this system. Elbel et al. [67] described the quality of spheroidal graphite iron and especially the formation of voids and holes as a result of reactions occurring during casting operations. The impact of process parameters on the formation of defects was also assessed in this work. Unfortunately, no identical terms were found in this work for the voids interchangeably referred to in the text, it was impossible to relate these defects to any classification, which greatly hinders the understanding process. In Europe, there are classification systems for casting defects in France, England, Germany, and Poland. Classification schemes of defects in these castings are presented in Figure 4.

In Poland, there is a division [64] in which two levels are distinguished (flow A). At the upper level, 4 defect groups are identified. At the lower level, each group is assigned defects with specific features. These defects were given names that help unambiguously identify them.

In the French foundry industry, a multi-level structure is used [58,60,61,63] whose first level contains seven groups named, respectively:

- Outer metal gain;
- Inner and outer cavities;
- Breaks in casting continuity;
- Surface defects;
- Incompleteness of the product;
- The inaccuracy of dimensions or shape;
- Structural inclusions or anomalies.

The lowest level also contains the names of individual defects, but between this level and the defect group definition, there are two intermediate levels containing additional features of a given group or subgroup (flow B). This way, each defect is assigned certain characteristics to make it easier to identify its causes and take preventive actions [58,60,63].

In the English and German literature, defects are classified in a different manner [56,57,63]. The classification of defects in accordance with BS 2737: 1956 [56,57,60,61,62,63] includes terminology of internal casting defects identified by ultrasonic flaw detection.

The principle of this division is presented in Figure 4 (flow C). The names of defects are presented directly here, and they are assigned to cause groups and the particular causes of their formation. It includes a defect atlas illustrated with 65 reproductions of radiographs or micrographs. This division would be very convenient for identification, but some causes were defined somewhat inaccurately. Since several defects may have a common cause, this division may not always be used objectively. A proposal for systematics of defects was also presented by Guy [60], and this paper was inauspiciously translated as “Atlas of Casting Defects” [61] and presented as a work resulting from French-German cooperation. It also adopts a division into seven classes marked with letters:

- Metallic growth;
- Cavities;
- Breaks in continuity;
- Surface defects;
- Incomplete casting;
- Incorrect dimensions or shapes;
- Inclusions or abnormal structure.

Each class is divided into groups, and those in turn into subgroups marked with letters, a given defect is determined by a letter and a number, which makes it possible to position it in the classification. However, the names of defects vary depending on geographical regions and depend on the people using the classification, which prevents international polemics and communication. It is identical in the French and German versions. This classification is a very valuable collection of information; however, the multitude of markings and the lack of unambiguous terms introduce some chaos and raise some controversy among cast manufacturers. It is a great tool for scientists, but it is too complicated and difficult to read for employees who are the basic staff of a foundry.

Kassie et al. characterized steel casting defects in their project. Unfortunately, they only provided a detailed description of two defects of castings from these materials, which in their opinion have the greatest impact on the quality of the product, i.e., gas defects and shrinkage defects [56].

Noteworthy is the paper of Garat et al., who, in the first part of their paper, classified defects into eight groups, i.e., incorrect shapes—core and pin offset, incorrect dimensions, defective surfaces, discontinuity (cracking), metal growths, cavities, inclusions, typical defects of rheocasting in the semi-solid state (formes incorrectes—déport des noyaux et des broches, dimensions incorrectes, surfaces défectueuses, solution de continuité (fissuration), excroissances métalliques, cavités, inclusions, défauts typiques du rhéomoulage à l’état semi-solide) [62]; however, this paper applies only to aluminum alloys. Interest in aluminum alloys in recent years is due to their prevalence [62,67,68,69,70,71,72,73,74], thanks to their low density combined with good mechanical and corrosive properties. In 2015, Fiorese, et al. created a new classification for defects in aluminum alloy castings [74]. This work proposes a multi-level classification of structural defects. The first level distinguishes defects based on their location (internal, external or geometric), the second level distinguishes defects based on their metallurgical origin.

**Figure 5:**
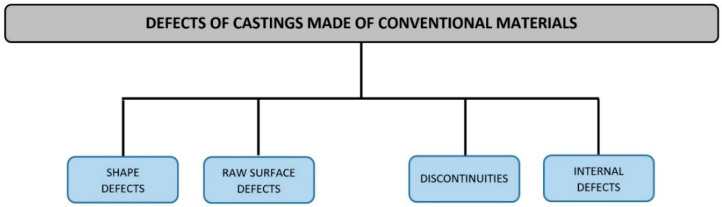
Defects of castings from traditional materials (steel, cast iron, non-ferrous alloys).

Polish classification of defects in metal castings is one of the few classifications covered by governmental standards. As can be seen in Figure 4, this classification (flow A) is the simplest because of its two-stage arrangement. Its additional feature is a clear division of defects in castings made from different materials. The features specified as well as the wide availability and familiarity of this classification in Poland make it necessary to refer to this classification while attempting to create any other classification. Thus, further considerations will be based on the distribution of defects contained in it. For this reason, a more detailed description of this classification is needed. According to the Polish standard [64] as well as national studies, e.g., atlases [13,61], this classification contains four groups of defects, listed in Figure 5.

The order of defect groups is consistent with the sequence of operations in the casting acceptance by the quality control department. Shape defects are observed first, followed by raw surface defects, and discontinuities. Internal defects are detected during non-destructive and destructive testing as well as during machining of castings. Each of the four groups is assigned certain defects, which are marked with “W”, and the type of material in which they occur is indicated in Table 1.

The Polish Standard (PN-85/H-83105) also provides indicative causes of defects. In other studies [13,53,54,55,57,59], which properly extend this standard, methods of detection of defects formed were also found.

## 4. Classification of Defects in Metal Composite Castings and a Proposal for the Division of Defects in Castings Made from Traditional Materials (Cast Steel, Cast Iron, Non-Ferrous Alloys) and Composite Materials

**Figure 6:**
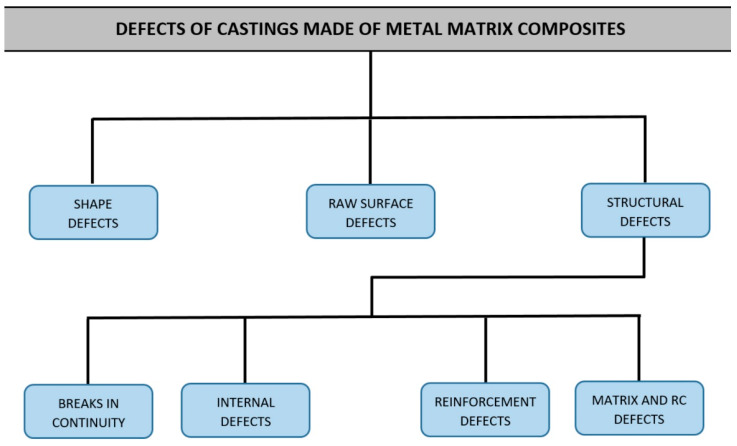
Structure of classification of defects in metal composite castings.

This paper proposes a classification of defects in the structure of metal composite castings, which is a group in the classification of casting defects. This group is called structural defects. Other groups of defects (defects in shape and defects in the raw surface) covered by the classification correspond to the groups appearing in the classification of defects in castings made from traditional materials (according to PN-85/H-83105—Figure 5). This group (structural defects) consists of four subgroups covering the defects in the structure of castings made from traditional materials, which, at the same time, correspond to the defects in the structure of composite castings (Figure 6).

The classification of defects in the structure of metal composite castings is presented in Table 2. The layout of the table is similar to that in the Polish Standard [64]. It presents the qualification of a defect into the appropriate subgroup of defects in the structure of metal composite castings, as is described; the scheme is supported by an example of the defect, the causes of defects in the structure of metal composite castings are presented based on the studies [1,2,3,28,32,33,47] and their detection methods in accordance with studies of the authors [1,39,40,41,42,43,44,75,76,77,78,79,80,81,82,83,84,85,86,87,88,89,90,91,92,93,94,95,96] is proposed. Table 3 presents a proposal for the division of defects in castings made from traditional materials (cast steel, cast iron, non-ferrous alloys) and composite materials compatible with standard PN-85/H-83105 [67]. The nomenclature for marking a specific defect (numbering), in the case of composite casting defects, has been supplemented by the author’s proposition according to the key: W-Group-Subgroup-Sequential No. For example, W-400-32-1 means the first defect (1) of the type “Inclusions” (400) classified into group 3 (structural defects), subgroup 2 (internal defects).

## 5. Open Atlas of Casting Defects Project

Computer-aided production processes [84,85,97,98], including casting as well as an assessment of the quality of castings using computer software for their diagnostics is a constantly developing field of science [86,87,88,89,90]. From the point of view of casting production, wide, quick access to industry information, databases, standards, consulting and technological descriptions is very important. Decision support and diagnostics, in the case of foundries, allow for the use of software tools and computer hardware of various types [63,88,89,90]. An example of such an application can be the study of Kluska-Nawarecka, which focuses on the problems related to creating formal descriptions of knowledge about defects. It presents the methodology of creating models using neural networks and genetic algorithms in the field of defect diagnostics [63]. El-Tokhy et al. developed digital control of casting defects using radiographic X-ray images [76]. In this work, an artificial neural network is used as a classifier to match the objectives of the identified product features, and three different algorithms introduced automatically to detect casting defects on X-rays. Automation of the diagnostic process is not only conducive to improving the quality of the product, it also facilitates control and improves the manufacturing process, and increases efficiency and profitability by reducing labor costs [63,69,89,90,98]. Mery et al., on image processing to detect damage in aluminum and steel castings, described the main aspects of the automatic unit for X-ray control of products [69]. Perzyk [97] characterized the main possibilities and potential applications of data mining in the casting manufacturing industry. This work identifies the main types of data mining techniques, including statistics, artificial intelligence, databases, and visualization tools. Statistical methods and visualization methods are presented in more detail, showing their general capabilities and advantages as well as characteristic examples of applications in foundry production.

All the above-mentioned premises, as well as the development of the author’s classification of casting defects based on the standard [64], which also takes defects of castings from metal composites into account, prompted the authors of the article to develop the Open Atlas of Casting Defects (OACD). It is a continuation of the authors’ paper [1,54,55,98,99,100] and ultimately aims to provide an open access platform for casting manufacturers, their customers and researchers involved in the assessment of casting defects and the origin of their formation. The open access formula will allow users to modify and complement data, and constantly update knowledge of castings and their defects. The rest of the chapter presents the capabilities and functions of the Open Atlas of Casting Defects, whose working draft is designed in Excel and Visual Basic for Applications (VBA). Ultimately, it will be a web-based expert program based on the follow-up system using modern internet technologies, such as: HTML 5.0, PHP, Java, Ajax.

**Figure 7:**
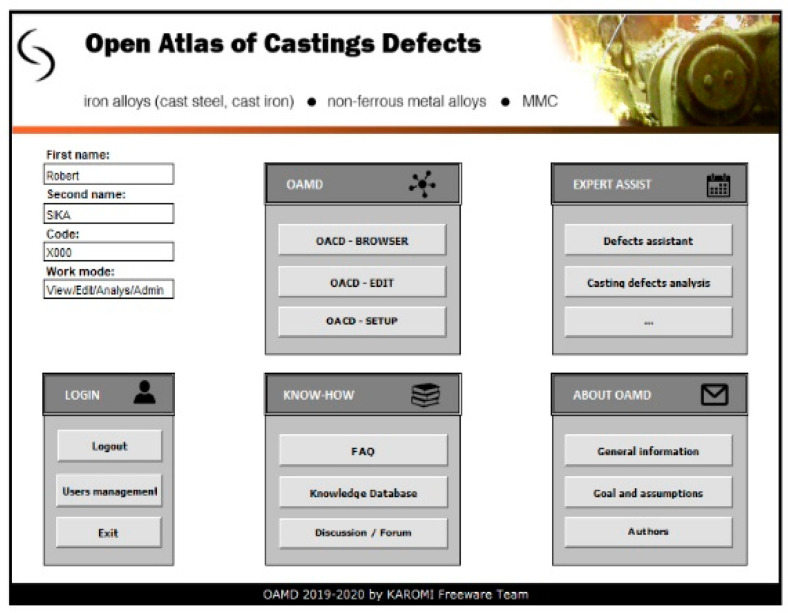
The main window of the Open Atlas of Casting Defects.

The main window of the program (Figure 7) allows you to choose the method of working with the Open Atlas of Casting Defects. In addition to typically administrative functions related to login, privileges and settings of the atlas, the user has the possibility to:

- View the atlas of defects containing casting defects and their detailed description (view mode, code C001);
- View casting defects’ classifications (view mode, code C001);
- Search for casting defects using many different criteria (view mode, code C001);
- Edit the knowledge base—modify and complement information on casting defects (edit mode, code B001);
- Obtain expert advice (analysis mode, code A001);
- Use the forum to exchange experience and knowledge (analysis mode, code A001);
- Open atlas of casting defects management (admin mode, code X001).

**Figure 8:**
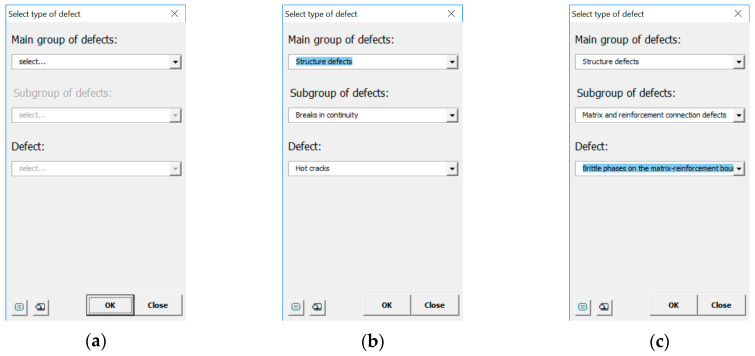
Open Atlas of Casting Defects: (**a**–**c**) searching for a defect in the window containing the classification of defects in castings made from traditional materials and composite materials.

**Figure 9:**
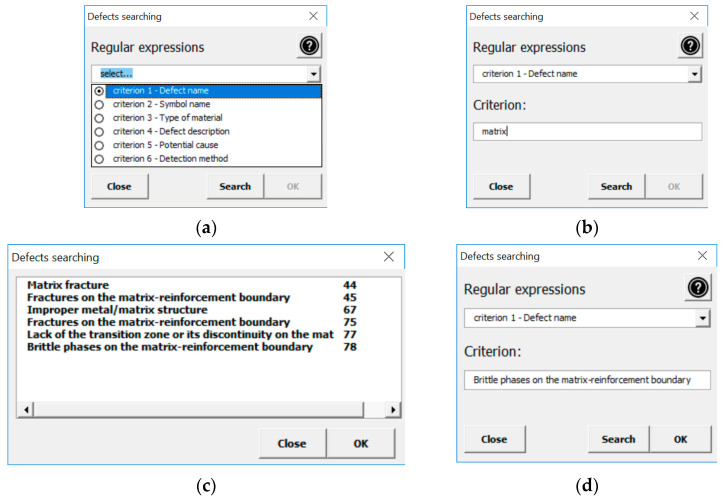
Open Atlas of Casting Defects: (**a**–**d**) searching for a defect in the window containing the search criteria.

The user can access descriptions of individual defects in various ways, depending on their needs. On the one hand, the user can search for a given defect using the drop-down menus in the window presenting the classification of defects (Figure 8). It contains defects of castings made from traditional materials (cast steel, cast iron, non-ferrous alloys) and composite materials. By choosing individual options, in several steps through groups of defects and their subgroups, the user “arrives at” the defect that interests them and its description. On the other hand, it is possible to search for a given defect using the criteria available in the program (i.e., Defect Name, Marking, Occurrence, Defect Description, Alleged Cause, Detection Method). The user, knowing the name or cause of the defect, can search for the defect that interests them. As the research and experience of the authors based on conducted work in industry show, this top-down approach with the possibility of multi-criteria search is very desirable for quality controllers. Figure 9 shows how to search for a specific defect, taking its name into account.

**Figure 10:**
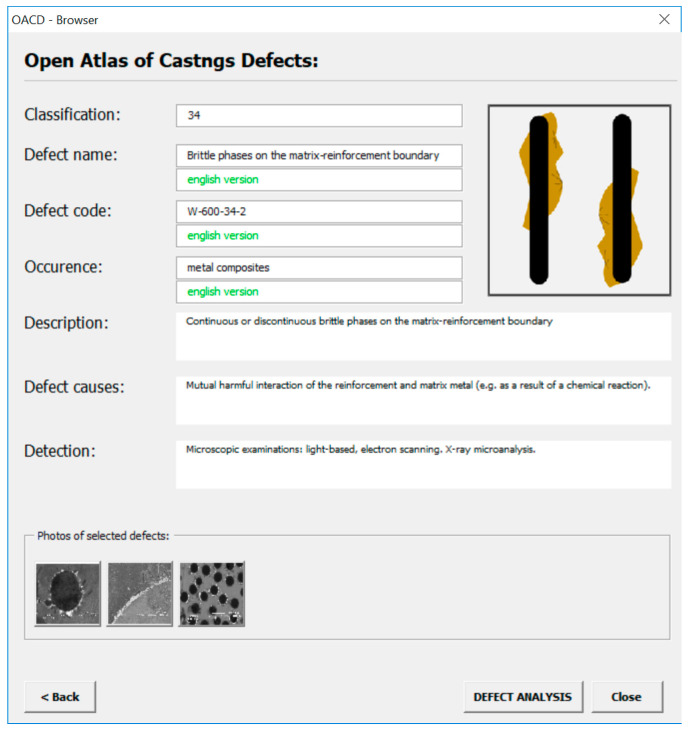
The window containing a detailed description of the defect.

The most important element of the Open Atlas of Casting Defects is the knowledge base containing a detailed description of casting defects. A representative of this knowledge base in the form of a program window is shown in Figure 10 for the defect: “Brittle phases on the matrix-reinforcement boundary”. The user can obtain information about:

- Qualification of the defect;
- Name/names of the defect—this field contains valid/acceptable/common names of the given defect;
- Defect code—the code assigned to the classification of a defect of castings made from traditional materials (cast steel, iron, non-ferrous alloys) and composite materials on which the Open Atlas of Casting Defects is based;
- Occurrence in a given material group—is the defect present in castings made from traditional or composite materials;
- Description of the defect that occurred;
- Causes of the defect;
- Defect detection method.

**Figure 11:**
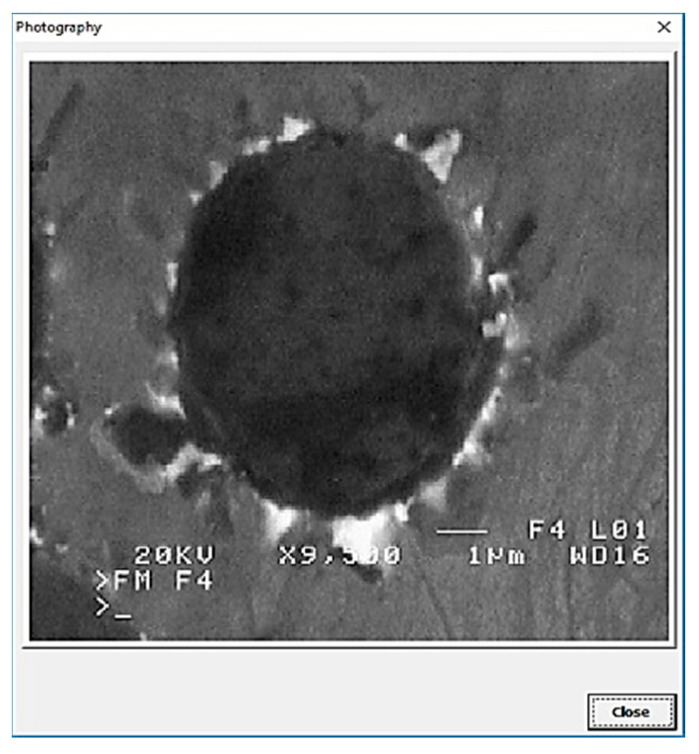
The window containing a picture of the defect.

In addition to the verbal description of casting defects, each defect is also accompanied by its scheme (cf. Figure 10) and picture (Figure 11) (pictures of actual casting defects updated on a regular basis for various produced series shown on the casting fragments for defects visible on the surface and on properly cut and prepared samples for internal defects).

## 6. Conclusions

The results of analyses and theoretical findings, as well as experimental research presented in the paper, allow to draw the following conclusions:

1. The classification of structure defects appearing in castings of cast metal composites proposed in the paper allows to:

- Complement the classification of defects in castings of traditional materials, with the group of defects characteristic for castings being a concern of this paper;
- Unequivocally define defects characteristic for castings of studied composites;
- Supplement the proposed classification with possible defects not taken into consideration thanks to its open character;

2. Determining the causes of defects covered by the classification as a result of analysis of the process of manufacturing castings made from tested composites is facilitation in undertaking actions aimed at eliminating these defects;

3. The presented classification of defects in metal composite castings complemented by the causes of their formation as well as methods of their detection and identification may constitute the starting point for the development of an expert program, which would support undertakings related to quality control.

## Figures and Tables

**Table 1:** Classification of defects in castings from traditional materials (own study according to the Polish Standard [64]).

Defect Name	Marking	Occurrence
	**Group 1—Shape Defects**	
Mechanical damage	W-101	all alloys
Misrun	W-102	all alloys
Knob	W-103	all alloys
Flash	W-104	all alloys
Mismatch (shift)	W-105	all alloys
Swelling	W-106	all alloys
Warping	W-107	all alloys
	**Group 2—Raw Surface Defects**	
Roughness	W-201	all alloys
External bubble	W-202	all alloys
Pitted skin	W-203	cast steel
Pock-marking	W-204	all alloys
Pinholes	W-205	all alloys
Shrinkage depression	W-206	all alloys
Cold lap	W-207	all alloys
Sand buckle	W-208	all alloys
Rat tails	W-209	all alloys
Sand holes	W-210	all alloys
Crush	W-211	all alloys
Contamination	W-212	all alloys
**Defect Name**	**Marking**	**Occurrence**
Scale	W-213	malleable cast iron
Galling	W-214	non-ferrous metals
Partial melting (during annealing)	W-215	malleable iron cast
Elephant skin	W-216	spheroidal graphite iron
Sweat	W-217	non-ferrous metals
Flowers	W-218	non-ferrous metals
Metal penetration	W-219	all alloys
Veins	W-220	all alloys
Burning-on (of sand)	W-221	all alloys
Sand holes	W-222	all alloys
Oxidation	W-223	non-ferrous metals
Peel	W-224	malleable cast iron
	**Group 3—Discontinuities**	
Hot cracks	W-301	all alloys
Cold cracks	W-302	all alloys
Shrinkage cracks	W-303	all alloys
Annealing cracks	W-304	malleable cast iron
Transgranular cracks	W-305	cast steel, non-ferrous metals
	**Group 4—Internal Defects**	
Gas bubble	W-401	all alloys
Gas bubble	W-401	all alloys
Porosity	W-402	all alloys
Shrinkage cavity	W-403	all alloys
Microporosity	W-404	all alloys
Slag inclusion	W-405	all alloys
Sand drops	W-406	all alloys
Cold shots	W-407	all alloys
Foreign metal	W-408	all alloys
Segregation	W-409	non-ferrous metals
Coarse-grained structure	W-410	non-ferrous metals
Hard spots	W-411	cast iron
Grey spots	W-412	malleable cast iron
White fracture	W-413	malleable cast iron
Bright fracture	W-414	malleable cast iron
Bright border	W-415	malleable cast iron
Heterogeneity	W-416	all alloys

**Table 2:** Classification of defects in the structure of metal composite castings (description of defects in metal composites proposed by the authors).

Symbol, Name, (Description)	Pictures and Scheme, Additional Description	Probable Causes	Detection or Identification Methods
SUBGROUP 3.1—Breaks in Continuity			
Fractures of reinforcement elements (FRE) (break, crack, lack of continuity of reinforcement elements).	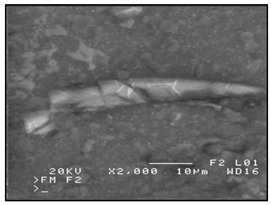 **A1.** SEM image of the clearly visible damages of the reinforcing phase in an ex situ composite, produced by saturating the reinforcement with liquid matrix (composite: silumin/Al_2_O_3_/SiO_2_). 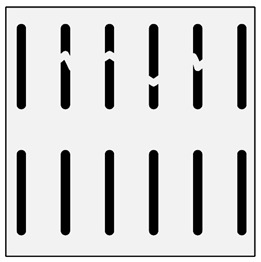 **A2.** Scheme of fractures of reinforcement elements.	Improper manufacturing process too high, saturation pressure or the flow velocity of matrix metal. Internal stresses inside the casting, too intense mould cooling, too late removal of the casting from the mould, abrupt cooling of the casting after removing it from the mould.	Microscopic examinations (light-based, electron scanning).
[MF] Matrix fracture (MF) (breaks in matrix material continuity located most frequently around the reinforcing phase).	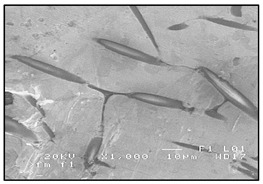 **B1.** SEM image of the irregularly shaped breaks in the matrix structure in an ex-situ composite, produced by saturating the reinforcement with liquid matrix (composite: silumin/Al_2_O_3_/SiO_2_). 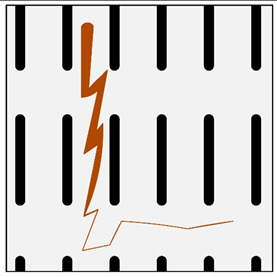 **B2.** Scheme of matrix fracture.	Internal stresses inside the casting caused by improper mould construction, improper mould temperature, too intense mould cooling, too late removal of the casting from the mould.	Microscopic examinations (light-based, electron scanning).
Fractures on the matrix-reinforcement boundary (FMRB) (no connection between the matrix and reinforcement).	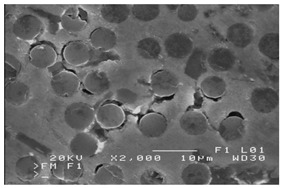 **C1.** SEM image of the visible breaks in continuity between the reinforcement and the matrix in an ex-situ composite, produced by saturation of the reinforcement with the matrix (composite: silumin/long carbon fibre). 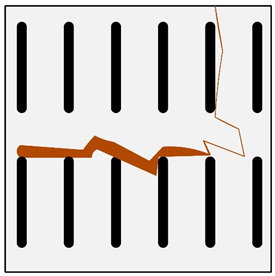 **C2.** Scheme of fractures on the matrix-reinforcement boundary.	Internal stresses inside the casting, improper mould temperature, too intense mould cooling, too late removal of the casting from the mould, abrupt cooling of the casting after removing it from the mould.	Microscopic examinations: (light-based, electron scanning).
**SUBGROUP 3.2—Internal Defects**			
Inclusions (INC) (precipitations of chemical composition and structure different from the matrix or reinforcement).	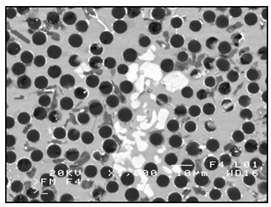 **D1.** SEM image of the inclusions in the composite structure, visible bright spots between the reinforcing phase—black circles, in an ex situ composite, produced by saturation of the reinforcement with the matrix (composite: silumin/long carbon fibre). 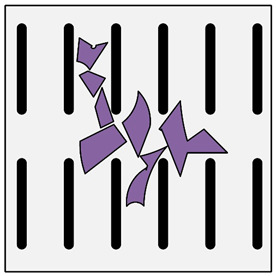 **D2.** Scheme of inclusions.	Impure charge material, improper chemical constitution. Improper process of melting and refining. Erosion of mould or its coating, caused by using wrong materials or too high flow velocity of matrix metal.	Microscopic examinations: (light-based, electron scanning). Ultrasound and radiography flaw detection.
Unfilled reinforcement spaces (URS) (free spaces in reinforcement and matrix contact zones).	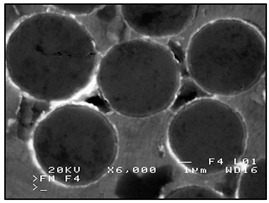 **E1.** SEM image of the unfilled reinforcement spaces due to, e.g., insufficient saturation in the ex-situ composite, produced by saturation of the reinforcement with the matrix (composite: silumin/long carbon fibre). 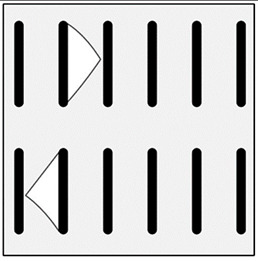 **E2.** Scheme of unfilled reinforcement spaces.	Improperly conducted manufacturing process, insufficient wetting of the reinforcement by the matrix. Too low saturation pressure, too low temperature of the reinforcement, matrix metal or mould.	Microscopic examinations (light-based, electron scanning). Gravimetric examination.
Occluded gas bubbles (OGB) (pores of a shape close to spherical, present in whole volume of the casting, with increased dimensions in isolated areas of the casting).	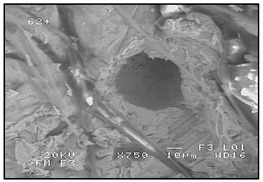 **F1.** SEM image of the occluded gas bubbles, in an ex situ composite, produced by saturating the reinforcement with the matrix (composite: silumin/short fibre Al_2_O_3_/SiO_2_). 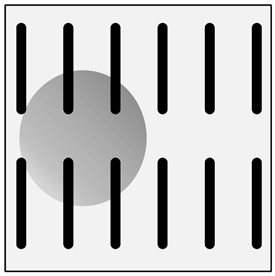 **F2.** Scheme of occluded gas bubbles.	Improperly conducted manufacturing process, e.g., improper feed of matrix metal, too high velocity of saturation, too low temperature of reinforcement, matrix metal or mould, improper venting of the mould.	Microscopic examinations (light-based, electron scanning). Gravimetric examination Ultrasound and radiography flaw detection. Macroscopic examination. Computed tomography. Computer image analysis.
Separated (precipitated) Precipitate gas bubbles (SGB) (gas bubbles of a regular spherical shape, usually located on reinforcement elements).	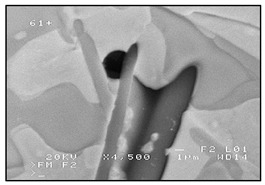 **G1.** SEM image of the precipitate gas bubbles located on the reinforcement fibre, in an ex situ composite, produced by saturation of the reinforcement with the matrix (composite: silumin/short fibre Al_2_O_3_/SiO_2_). 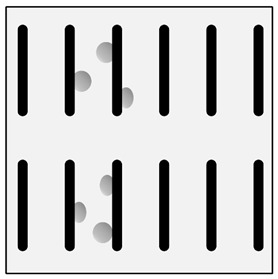 **G2.** Scheme of separated (precipitated). Precipitate gas bubbles.	Impure charge material, improperly prepared matrix material, improper melting or refining, improper preparation of reinforcement material.	Microscopic examinations (light-based, electron scanning). Gravimetric examination. Ultrasound and radiography flaw detection. Macroscopic examination. Computed tomography. Computer image analysis.
Improper metal/matrix structure (IMMS) (foreign phases, undesirable dendritic structure, presence of phases of variable chemical constitution, coarse grain structure).	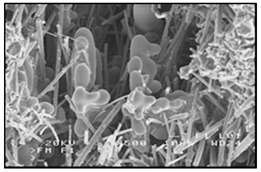 **H1.** SEM image of the incorrect matrix structure, dendrites of α phase in the structure of castings, formed on reinforcement fibres, produced by saturation of the reinforcement with the matrix (composite: silumin/short fibre Al_2_O_3_/SiO_2_). 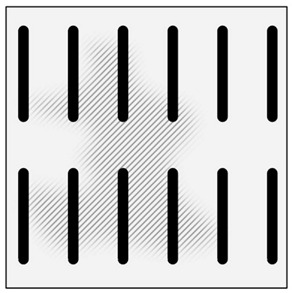 **H2.** Scheme of improper metal/matrix structure.	Too high or too low temperature of the matrix metal, improper chemical constitution of the matrix metal, improper modification or lack thereof, improper mould temperature.	Microscopic examinations (light-based, electron scanning). Computer image analysis. X-ray microanalysis.
**SUBGROUP 3.3—Reinforcement Defects**			
Inhomogeneity of size of reinforcement elements (ISiRE) (diversified size of the reinforcing phase).	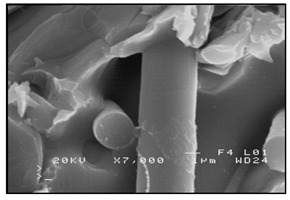 **J1.** SEM image of the inhomogeneity of size of reinforcement elements in the form of different sizes of reinforcement sections—black circles, in an ex-situ composite, produced by saturation of the reinforcement with the matrix(composite: silumin/short fibre Al_2_O_3_/SiO_2_). 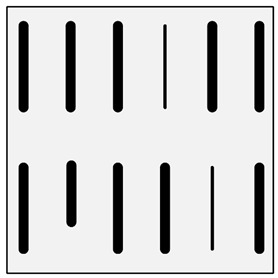 **J2.** Scheme of inhomogeneity of size of reinforcement elements.	Bad quality of the reinforcement.	Microscopic examinations (light-based, electron scanning). Computed tomography. Computer image analysis.
[IDRE] Inhomogeneity of distribution of reinforcement elements (IDRE) (varied density of the reinforcing phase in different areas of the reinforcement).	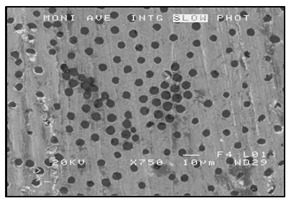 **K1.** SEM image of the clearly visible uneven distribution of reinforcing elements in the studied area, in an ex-situ composite, produced by saturation of the reinforcement with the matrix (composite: silumin/long carbon fibre). 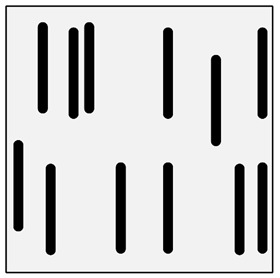 **K2.** Scheme of inhomogeneity of distribution of reinforcement elements.	Bad quality of the reinforcement, overheating of the composite suspension, failure to mix the suspension, which may lead to sedimentation of the reinforcing phase particles, matrix contamination.	Microscopic examinations (light-based, electron scanning). Computed tomography Computer image analysis.
Foreign matter in the reinforcement structure (FMRS) (impurities formed during reinforcement manufacturing).	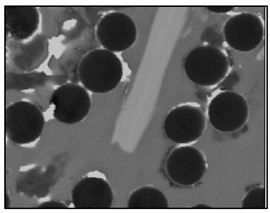 **L1.** SEM image of the foreign matter in the reinforcement structure, identified fragment of another metal—iron in the composite structure. An ex situ composite, produced by saturation of the reinforcement with the matrix (composite: silumin/long carbon fibre). 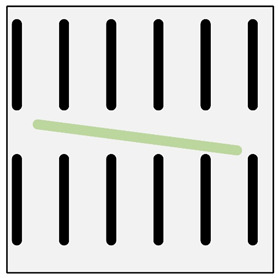 **L2.** Scheme of foreign matter in the reinforcement structure.	Bad quality of the reinforcement. Impure charge material. Improperly prepared matrix material, improper melting or refining, improper preparation of reinforcement material.	Microscopic examinations (light-based, electron scanning). X-ray microanalysis.
Deformation of the reinforcing structure (DTS) (improper shape of reinforcement structure. This defect occurs only in the group of an ex-situ saturated composites).	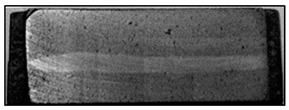 **M1.** Macroscopic image of the deformed bright area of the reinforcing moulder in an ex-situ composite, produced by saturating the reinforcement with the matrix (composite: silumin/short fibre Al_2_O_3_/SiO_2_). 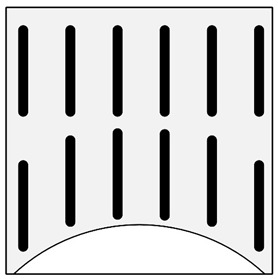 **M2.** Scheme of deformation of the reinforcing structure.	Mechanical damage to the reinforcement moulder. Too high pressure or velocity of matrix metal flow.	Macroscopic examination. Radiography flaw detection. Ultrasonic flaw detection.
Improper localization of the reinforcing structure (ILRS) (displacement of the reinforcement structure in the casting space. This defect occurs only in the group of an ex-situ saturated composites).	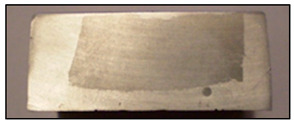 **N1.** Macroscopic image of the unevenly distributed reinforcing moulder in an ex situ composite, produced by saturating the reinforcement with the matrix (composite: silumin/short fibre Al_2_O_3_/SiO_2_). 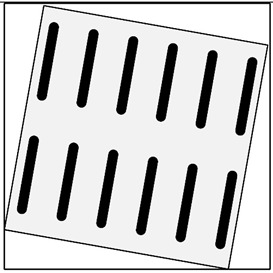 **N2.** Scheme of improper localization of the reinforcing structure.	Improper preparation of the mould, ensuring stable placement of the moulder, too high metal pressure which results in a shift in the reinforcement structure in the mould.	Macroscopic examination. Radiography flaw detection. Ultrasonic flaw detection.
**SUBGROUP 3.4—Matrix and Reinforcement Connection Defects**			
Lack of the transition zone or its discontinuity on the matrix – reinforcement boundary (LTZ-DMRB) (visible lack of appearance of longitudinal band which is a transitional zone differing in colour and chemical composition from the structure of reinforcement and matrix).	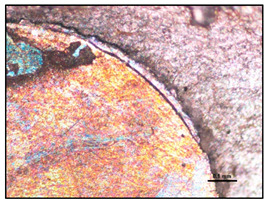 **O1.** Optical microscopy image of the transitional zone dark field on the boundary: matrix bright area, reinforcement grey-green area; an ex-situ composite produced by saturation of the reinforcement with the matrix (composite: silumin/steel fibre (Cr18Ni9)). 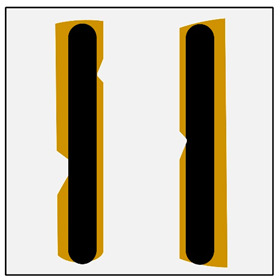 **O2.** Scheme of lack of the transition zone or its discontinuity on the matrix—reinforcement boundary.	Improper preparation of reinforcement, impurities of the reinforcement, e.g., failure to degrease.	Microscopic examinations (light-based, electron scanning). X-ray microanalysis.
Brittle phases on the matrix-reinforcement boundary (BP-MRB) (continuous or discontinuous brittle phases on the matrix-reinforcement boundary).	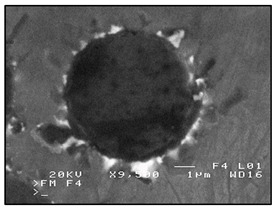 **P1.** SEM image of the reinforcement in the form of bright points around the reinforcement (black circle) in an ex-situ composite, produced by saturation of the reinforcement with the matrix (composite: silumin/long carbon fibre). 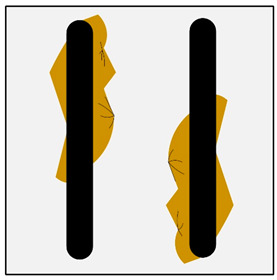 **P2.** Scheme of brittle phases on the matrix-reinforcement boundary.	Mutual harmful interaction of the reinforcement and matrix metal (e.g., as a result of a chemical reaction).	Microscopic examinations (light-based, electron scanning). X-ray microanalysis.

**Table 3:** Proposed division of defects in castings made from traditional materials (cast steel, cast iron, non-ferrous alloys) and all composite materials.

Defect Name	Marking	Occurrence, Castings:
**Group 1—Shape Defects**		
Mechanical damage	W-101	all alloys, all metal composites
Misrun	W-102	all alloys, all metal composites
Knob	W-103	all alloys, all metal composites
Flash	W-104	all alloys, all metal composites
Mismatch (shift)	W-105	all alloys, all metal composites
Swelling	W-106	all alloys, all metal composites
Warping	W-107	all alloys, all metal composites
**Group 2—Raw Surface Defects**		
Roughness	W-201	all alloys, all metal composites
External bubble	W-202	all alloys, all metal composites
**Defect Name**	**Marking**	**Occurrence, Castings:**
Pitted skin	W-203	cast steel
Pock-marking	W-204	all alloys, all metal composites
Pinholes	W-205	all alloys, all metal composites
Shrinkage depression	W-206	all alloys, all metal composites
Cold lap	W-207	all alloys, all metal composites
Sand buckle	W-208	all alloys, all metal composites
Rat tails	W-209	all alloys, all metal composites
Sand holes	W-210	all alloys, all metal composites
Crush	W-211	all alloys, all metal composites
Contamination	W-212	all alloys
Scale	W-213	malleable cast iron
Galling	W-214	non-ferrous metals
Partial melting (during annealing)	W-215	malleable iron cast
Elephant skin	W-216	spheroidal graphite iron
Sweat	W-217	non-ferrous metals
Flowers	W-218	non-ferrous metals
Metal penetration	W-219	all alloys, all metal composites
Veins	W-220	all alloys, all metal composites
Burning-on (of sand)	W-221	all alloys, all metal composites
Sand holes	W-222	all alloys
Oxidation	W-223	non-ferrous metals
Peel	W-224	malleable cast iron
**Group 3—Discontinuities**		
**Subgroup 1**—**Breaks in Continuity**		
Hot cracks	W-301	all alloys, all metal composites
Cold cracks	W-302	all alloys, all metal composites
Shrinkage cracks	W-303	all alloys, all metal composites
Annealing cracks	W-304	malleable cast iron
Transgranular cracks	W-305	cast steel, non-ferrous metals
Fractures of reinforcement elements	Own marking W-300-31-1	all metal composites
Matrix fracture	Own marking W-300-31-2	all metal composites
Fractures on the matrix-reinforcement boundary	Own marking W-300-31-3	all metal composites
**Subgroup 2—Internal Defects**		
Inclusions	Own marking W-400-32-1	all metal composites
Unfilled reinforcement spaces	Own marking W-400-32-2	all metal composites
Occluded bubbles	Own marking W-400-32-3	all metal composites
Separated gas bubbles	Own marking W-400-32-4	all metal composites
Gas bubble	W-401	all alloys, all metal composites
**Defect Name**	**Marking**	**Occurrence, Castings:**
Porosity	W-402	all alloys, all metal composites
Shrinkage cavity	W-403	all alloys, all metal composites
Microporosity	W-404	all alloys, all metal composites
Slag inclusion	W-405	all alloys, all metal composites
Sand drops	W-406	all alloys, all metal composites
Cold shots	W-407	all alloys, all metal composites
Foreign metal	W-408	all alloys, all metal composites
Segregation	W-409	non-ferrous metals
Coarse-grained structure	W-410	non-ferrous metals
Hard spots	W-411	cast iron
Grey spots	W-412	malleable cast iron
White fracture	W-413	malleable cast iron
Bright fracture	W-414	malleable cast iron
Bright border	W-415	malleable cast iron
Heterogeneity	W-416	all alloys, all metal composites
Improper metal/matrix structure	Own marking W-400-32-5	all alloys, all metal composites
**Subgroup 3—Reinforcement Defects**		
Inhomogeneity of shape of reinforcement elements	Own marking W-500-33-1	all metal composites
Inhomogeneity of size of reinforcement elements	Own marking W-500-33-2	all metal composites
Inhomogeneity of distribution of reinforcement elements	Own marking W-500-33-3	all metal composites
Foreign matter in the reinforcement structure	Own marking W-500-33-4	all metal composites
Deformation of the reinforcing structure	Own marking W-500-33-5	saturated metal composites
Improper localization of the reinforcing structure	Own marking W-500-33-6	saturated metal composites
Fractures on the matrix-reinforcement boundary	Own marking W-500-33-7	all metal composites
**Subgroup 4—Matrix and Reinforcement Connection Defects**		
Lack of the transition zone or its discontinuity on the matrix – reinforcement boundary	Own marking W-600-34-1	all metal composites
Brittle phases on the matrix-reinforcement boundary	Own marking W-600-34-2	all metal composites
